# Combining EEG signal processing with supervised methods for Alzheimer’s patients classification

**DOI:** 10.1186/s12911-018-0613-y

**Published:** 2018-05-31

**Authors:** Giulia Fiscon, Emanuel Weitschek, Alessio Cialini, Giovanni Felici, Paola Bertolazzi, Simona De Salvo, Alessia Bramanti, Placido Bramanti, Maria Cristina De Cola

**Affiliations:** 1 Institute of Systems Analysis and Computer Science A. Ruberti (IASI), National Research Council (CNR), Via dei Taurini 19, Rome, 00185 Italy; 2 SysBio Centre for Systems Biology, Rome, Italy; 3 Department of Engineering, Uninettuno International University, Corso Vittorio Emanuele II 39, Rome, 00186 Italy; 4 IRCCS Centro Neurolesi “Bonino-Pulejo”, Contrada Casazza, SS113, Messina, 98124 Italy

**Keywords:** Alzheimer’s disease, Feature extraction, Electroencephalography signals, Classification

## Abstract

**Background:**

Alzheimer’s Disease (AD) is a neurodegenaritive disorder characterized by a progressive dementia, for which actually no cure is known. An early detection of patients affected by AD can be obtained by analyzing their electroencephalography (EEG) signals, which show a reduction of the complexity, a perturbation of the synchrony, and a slowing down of the rhythms.

**Methods:**

In this work, we apply a procedure that exploits feature extraction and classification techniques to EEG signals, whose aim is to distinguish patient affected by AD from the ones affected by Mild Cognitive Impairment (MCI) and healthy control (HC) samples. Specifically, we perform a time-frequency analysis by applying both the Fourier and Wavelet Transforms on 109 samples belonging to AD, MCI, and HC classes. The classification procedure is designed with the following steps: (i) preprocessing of EEG signals; (ii) feature extraction by means of the Discrete Fourier and Wavelet Transforms; and (iii) classification with tree-based supervised methods.

**Results:**

By applying our procedure, we are able to extract reliable human-interpretable classification models that allow to automatically assign the patients into their belonging class. In particular, by exploiting a Wavelet feature extraction we achieve 83%, 92%, and 79% of accuracy when dealing with HC *vs* AD, HC *vs* MCI, and MCI *vs* AD classification problems, respectively.

**Conclusions:**

Finally, by comparing the classification performances with both feature extraction methods, we find out that Wavelets analysis outperforms Fourier. Hence, we suggest it in combination with supervised methods for automatic patients classification based on their EEG signals for aiding the medical diagnosis of dementia.

## Background

Dementia is a broad group of brain disorders leading to a cognitive impairment because of a gradual dysfunction and death of brain cells. The World Alzheimer Report 2015 has been estimated that 36 million people were living with dementia in 2010, nearly doubling every 20 years to 66 million by 2030 and to 115 million by 2050 [1]. Given the continuous growth of incidence of this illness, dementia represents one of the major plague for the modern society. The most widespread cause of dementia is the Alzheimer’s disease (AD), which involves serious memory loss, cognitive impairment, and behavioural changes. Thus, AD interferes with daily, social and professional functioning of patients, also affecting the daily life of their families [2]. The intermediate stage between the normal cognitive deficit due to aging and dementia is defined as Mild Cognitive Impairment (MCI). Several symptoms distinguish MCI, but the loss of memory is a risk factor to develop AD [3]. In Europe, only 50% of the patients with dementia receive a diagnosis by a specialist centre, and tests for dementia are carried out after the patient has already started showing symptoms and the disease has progressed [4]. Usually, the process for obtaining a clinical diagnosis for dementia of a patient is mainly based on the delivery of a questionnaire in order to assess its cognitive abilities. However, a timely diagnosis would facilitate care, reduce the progression of the disease, and improve the patient’s management to alleviate the burden. This might be achieved through a combination of diagnosis criteria and reliable biomarkers.

In the past years, significant progresses have been made to detect the early stages of dementia through biochemical, genetic, neuroimaging, and neurophysiological biomarkers such as Electroencephalography (EEG) [5–9]. EEG provides the electrical activity of the brain by tracking the connectivity of neurons in the recording sites of the scalp [10], processing it with milliseconds precision. The condition of the brain physiology can be inferred from the EEG signals recorded, and thus abnormalities can be identified through the detection of unusual frequency patterns [11]. Indeed, different rhythms with diverse frequency bands describe the activity of the brain and can be recorded by EEG. Among them, the main ones are *alpha* (8-13 Hz, 30-50 *μV* amplitude), *beta* (13-30 Hz, 5-30 *μV* amplitude), *gamma* (≥ 30 Hz), *delta* (0.5-4 Hz), and *theta* (4-7 Hz, ≥ 20*μV* amplitude).

Although it is characterized by a lower spatial resolution than other neuroimaging techniques, EEG provides high temporal resolution [12]. Moreover, EEG is non-invasive, ease and faster to use and able to differentiate severity of dementia at a lower cost than other imaging devices [13, 14]. Thanks to its reduced costs EEG can be easily implemented for population screening to detect pre-clinical biomarkers.

EEG signal analysis may provide useful indications of the patterns of brain activity and predict the stages of dementia [15, 16] because of its significant capacity to detect brain rhythm abnormalities, generally correlated with the severity of cognitive impairment [17]. In particular, different clinical studies confirm EEG as suitable technique to early detect AD [18–20], due to the following effects on EEG signals: reduction of the complexity, perturbation of the synchrony, and slowingdown of the rhythms [19, 21, 22]. The slowing of the rhythms in the EEG signals of subjects affected by AD can be explained by a gain of the activity in the theta and delta frequency ranges, and a reduction of the activity in the alpha and beta frequency ranges [23–26]. The reduction of complexity in the EEG temporal patterns can be explained by a modification of the neural network architecture observed in subjects affected by AD [27, 28] due to loss of neurons and functional interaction alteration which make the activity of the brain more predictable, more regular, and simpler than in healthy control samples (HC) [29]. Therefore, we can state that EEG signals related to healthy controls subjects can be distinguished from those ones of subjects affected by neurodegenerative diseases (e.g., AD) or other pathologies (e.g., epilepsy).

Nevertheless, AD and MCI subjects are characterized by a huge variability and thus discriminating artifacts and patterns similarities to physiological brain activity still remain a crucial issue. In this regard, EEG signal processing integrated with computational algorithms based on machine learning methods may contribute to a deeper comprehension of the disease and simplify the work of neurologists providing an additional tool to diagnose the stage of dementia [20, 30–33].

In this paper, we propose a procedure based on EEG-signal preprocessing and automatic classification with supervised learning methods, and its application to discriminate subjects belonging to AD, or MCI, or HC classes. This is an extension of a preliminary work [34] in which we processed an EEG data set composed of 49 AD, 37 MCI and 14 healthy controls subjects (HC) by means of a spectrum analysis based on the Fourier Transformation, and we automatically classified them with supervised machine learning methods. Here, we have increased the number of HC subjects of the data set to 23 in order to balance the number of samples for each category. We have also improved the EEG-signal preprocessing and spectrum analysis techniques through the application of the Wavelet Transform as an efficient method for noise reduction and feature extraction, obtaining a more reliable method to distinguish healthy from diseased subjects.

## Methods

**Fig. 1:**
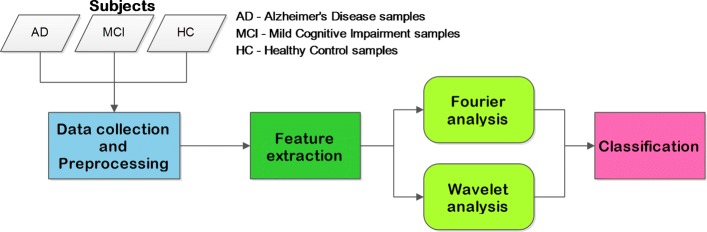
Flowchart of the EEG signal analysis procedure

We apply a procedure that encompasses the following steps summarized in Fig. 1: (i) data collection (subjects recruitment, EEG recording) and preprocessing, (ii) feature extraction (Fast Fourier and Wavelet Analysis), and (iii) classification (supervised machine learning to distinguish the AD, MCI, and HC classes).

### Data collection and preprocessing

**Subject Recruitment** The IRCCS Centro Neurolesi “Bonino-Pulejo” enrolled in 2012 and 2013 a total of 109 subjects: 86 patients affected by dementia (AD, MCI) of which 37 men and 49 women, and 23 healthy controls samples (HC) of which 13 men and 10 women. The patients have been classified either in AD or MCI, taking into account the World Health Organization standard. Subjects capable of undergoing an electroencephalogram and with a negative anamnesis for neurological comorbid disease have been included. Conversely, subjects under pharmacological treatment that could change the activity of the brain have been excluded from the study. Patients are mean aged 78.4 ± 6.4 and 74.1 ± 9.4 years, respectively for AD and MCI, whereas the mean age of healthy controls is 65.6 ± 7.9 years. Association between gender and etiological class (AD, MCI, HC) is not detected by the chi-square test (*p*-value > 0.05). Additionally, the difference in terms of age between men and women is not statistically significant according to the two-tailed Student’s T Test (p-value > 0.05 for each class). Thus, the hypothesis of homogeneity for age and gender among etiological classes cannot be rejected. Table 1 provides an overview of the enrolled subjects that can be divided in three main etiological classes: (i) patients with Alzheimer’s disease (AD), (ii) patients with Mild Cognitive Impairment (MCI), and (iii) healthy control samples (CT).

**Table 1:** Overview of the recruited subjects

Sample	Number of samples (%)			Average age (std dev.) in years		
type	Male	Female	Total	Male	Female	Total
AD	20 (41%)	29 (59%)	49	78.6 (4.1)	78.2 (7.6)	78.4 (6.4)
MCI	17 (46%)	20 (54%)	37	75.7 (9.7)	72.7 (9.1)	74.1 (9.4)
HC	13 (56%)	10 (44%)	23	68.1 (6.9)	62.3 (8.3)	65.6 (7.9)
Total	50 (46%)	59 (54%)	109	74.9 (8.2)	73.6 (9.9)	74.2 (9.1)

**EEG recording** We acquired multi-channel EEG signals by using 19 electrodes, by setting their placement according to the International 10-20 System [35], and by exploiting monopolar connections with earlobe electrode landmark [10]. The brain activity of the subjects in resting condition and closed eyes was measured in terms of electrical potential (*μV*). We recorded the EEG signals by capturing 300 seconds with 256 or 1024 sampling frequency (Hz).

**Preprocessing** For each signal we select the central 180 seconds (i.e., from 60 to 240 seconds) to avoid initial and final EEG recording artifacts. Additionally, to normalize the sampling frequency we convert each signal to 256 Hz.

### Feature extraction

Extracting features from EEG signals in frequency domain has been proven to be effective for analyzing the electrical brain activity with computational models [31, 36]. Thus, in order to obtain a set of informative features from the preprocessed EEG signals, we apply the Fourier and the Wavelet Transform for estimating their spectrum [37].

Firstly, the Fast Fourier Transform (FFT) is applied to each EEG signal of 180 seconds providing *M* Fourier Coefficients for each electrode (*M* equal to 16). Hence, for each sample we obtain 304 features (16 coefficients · 19 electrodes) and we arrange them in a matrix with 109 rows (referring to the samples) and 305 columns (304 referring to the features, and one referring to the sample type).

Secondly, the Discrete Wavelet Transform (DWT) is applied to each EEG signal of 180 seconds providing *M* Wavelet Coefficients for each electrode (*M* equal to 48). Hence, for each sample we obtain 912 features (48 coefficients · 19 electrodes) and we arrange them in a matrix with 109 rows (referring to the samples) and 913 columns (912 referring to the features, and one referring to the sample type).

We provide in Table 2 a schematic representation of the matrices.

**Table 2:** Schema of the matrix obtained after the feature extraction phase

Sample	Coefficient _(1,1)_	⋯	Coefficient _(*E,E*·*M*)_	Sample type
sample_1_	*a*_(1,1)_	⋯	*a*_(1,*E*·*M*)_	HC
sample_2_	*a*_(2,1)_	⋯	*a*_(2,*E*·*M*)_	MCI
⋯	⋯	⋯	⋯	⋯
sample _*N*_	*a*_(*N*,1)_	⋯	*a*_(*N,E*·*M*)_	AD

*N* = number of samples, *M* = number of coefficients, *M*+1 = number of features, *E* = number of electrodes, *a* = element of the matrix

The spectral analysis (Wavelet and Fourier) of the EEG signals has been performed by using the high level computing language provided by MATLAB ^*®*;^ R2014a [38].

#### Fourier analysis

We apply the Fast Fourier Transform (FFT) to obtain the spectrum of the EEG signals [37]. The FFT relies on the Discrete Fourier Transform (DFT) computed as follows:

1 $$ X [k] = \sum_{s=0}^{S-1} x[s]e_{k}[s]  $$

with *s* representing the s-*th* sample in the time domain; *x* corresponding to the signal time series (*s*=0,1,2,⋯,*S*−1); *X* referring to the representation of th frequency domain for the time-series signal *x*; *S* representing the the whole number of samples of the signal *x*; *k* corresponding to k-*th* frequency component (*k*=0,1,…,*S*−1); $e_{k}[s] = e^{-\frac {jks2\pi }{S}}$ referring to the k-*th* basis function.

*e*_*k*_[*s*] is calculated simultaneously during the sampling phase. Such a formula yields as output one complex number *X*[*k*] for each *k* component. The output of the FFT analysis are the Fourier Coefficients arranged in a matrix as shown in Table 2.

#### Wavelet analysis

A more effective way for decomposing time and frequency of the EEG signal, and for processing it is provided by the Wavelet Transform (WT). WT is a time-frequency representation of the signal, which is decomposed in different windows of variable size, i.e., sub-bands. Conversely to the FFT, the WT is able to catch the transient features of the analyzed signal [39], i.e., it enables to keep both the temporal (spatial duration) and frequency information of the signal. Indeed, WT allows to represent when transient events occur in the signal and with what intensity, as well as the time variations of the frequency contents [40]. Given a signal, WT decomposes it in simpler oscillating functions called wavelets. A family of wavelets (*ψ*_*a,b*_(*t*)) are derived from a unique mother wavelet *ψ*(*t*) by scaling (dilating and contracting) and by shifting it to different time positions [40, 41].

2 $$ \psi_{a,b}(t)= \frac{1}{\sqrt{\vert a \vert}}\psi \left(\frac{t-b}{a}\right)  $$

In Eq. 2*t* is the time variable, $a \in \mathbb {R}\setminus {0}$ is the scaling parameter, and *b*∈*R* is the shifting parameter. The wavelets are localized in both time and frequency with respect to the sinusoidal waves of Fourier, which are better localized in frequency, but infinitely extended in time [40]. Additionally, the former are limited in band, i.e., they are composed of a defined range of frequencies.

When dealing with digital signals that are frequency band-limited, the continuous form of WT can be discretized according to the sampling theorem [42]. The Discrete Wavelet Transform (DWT) allows to process digital signals by keeping enough information in reasonable computational time. A relevant feature of the DWT is the combination with high and low pass filters, through which the signals can be processed to filter the high and low frequencies in order to compress and reduce the noise [43], e.g., hidden artifacts and background noise during the EEG signals recording. Indeed, the WT is a well-established signal representation and feature extraction technique for EEG processing [44].

In this work, we adopt the DWT in order to perform the spectral analysis on the previously described dataset (see section Data collection and preprocessing). The choice of a simple DWT stems from the need of obtaining good performances over an arbitrary number of feature elements per channel and from the sampling frequency of the input signals (256 Hz). We adopt two types of discrete wavelet families: Daubechies (db) and Symlets (sym). Daubechies are compactly supported orthonormal wavelets [45], while Symlets are symmetrical wavelets proposed by Daubechies as modifications to the db family [46].

Given a single set of signals, each one is processed according to a feature extraction procedure composed of two main phases: noise reduction and feature extraction. Firstly, we perform a noise reduction phase, where each EEG signal is decomposed in *n* levels (i.e., sub-bands) by applying a DWT (Symlets order 3 wavelet type). For every sub-band *x* an upper and lower threshold value is calculated as:

3 $$\begin{array}{*{20}l}  {Thr}_{up}(x) = avg(x) + 1.5 \cdot stdev(x) \end{array} $$

4 $$\begin{array}{*{20}l}  {Thr}_{dwn}(x) = avg(x) - 1.5 \cdot stdev(x) \end{array} $$

The values of each sample *s*_*i*_ are then compared according to the defined thresholds (3) and (4) and if *s*_*i*_>*Thr*_*up*_ or *s*_*i*_<*Thr*_*dwn*_ then *s*_*i*_ is reduced as follows: *s*_*i*_∗(*Thr*_*up*_(*x*)−*Thr*_*dwn*_(*x*))/100. This step is performed in order to obtain an effective artifact reduction and to avoid possible information loss. The artifact removal phase operates on two levels of signal decomposition: level 5 and level 8. We choose these decomposition levels, because their ranges take into account the alpha, theta, beta, and delta bandwidths, which are widely adopted for EEG analysis and have been proven to be effective when dealing with Alzheimer’s diseased patients (see “Background” section for more details). The channel signal is then reconstructed with the obtained values, which are given as input to the feature extraction phase.

Secondly, for extracting the features, we adopt the Daubechies order 4 (db4) wavelet type with a sampling frequency of 256 Hz at decomposition level 5, which has been shown to guarantee a precise feature extraction in the brainwaves frequencies [47], and we perform a large set of test with different parameters obtaining lower performances. The feature extraction phase extracts the following statistical features: mean, standard deviation, and power spectral density of the wavelet coefficients. All the three feature types, representing the frequencies distribution of the EEG signals, are calculated over the *n* epochs of a channel-related signal. This phase makes use of the decomposition levels obtained by applying the DWT to the values produced during the noise reduction phase. Our method allows to apply an adaptive, threshold-based noise/artifact removal to the main bandwidths (i.e., alpha, theta, beta, delta). We extract 16 features per channel when considering the combination of only two bandwidths, i.e., alpha - theta or beta - delta, and 12 features per channel when taking into account all the four brainwaves.

The output of the DWT analysis are the Wavelet Coefficients arranged in a matrix as shown in Table 2.

### Classification

We perform a supervised learning analysis in order to automatically classify the samples to their types (HC, MCI, AD) by processing their associated features [48, 49]. Supervised learning automatically assigns a sample into a class by inferring a classification model from labeled data (training set). Our aim is to extract a human readable model specific for each type (HC, MCI, AD) of sample containing a small subset of features, e.g., ‘if *Wavelet*_10_>0.3 and *Wavelet*_16_<0.6 then the sample can be classified as MCI”). This model can support clinicians to identify pivotal features related to the investigated neurodegenerative disease and to diagnose new cases. In particular, we address the following classification problems: (i) HC *vs* AD; (ii) HC *vs* MCI; (iii) MCI *vs* AD; (iv) HC *vs* CASE (MCI+AD), where the CASE class is composed of AD joint to MCI samples in order to test the recognition of the diseased patients with respect to the healthy ones. Among the plethora of classification methods we use Decision Trees classifiers (i.e., C4.5 [50]), because they allow to handle noisy datasets and over-fitting with an ad-hoc parameters tuning. Additionally, Decision Trees provide the investigator with a compact, clear, and human readable classification model. C4.5 is an algorithm for the generation of decision trees used for classification. A decision tree is a structure similar to a flow chart, where each node denotes a test on an attribute, each branch represents a result of a test, and every leaf is labeled by a class. Indeed a node with outgoing edges is termed test node and the final nodes are the leaves.In decision trees the classification model permits to predict the class of a sample based on its features. The algorithm takes as input a set of classified data (training set) and the output is composed by leaf nodes, which define the belonging to a class attribute. Indeed, the path from the root to a specific leaf means that all the predicates applied to the features of the sample are verified. The validity of the three is verified on a set of labeled samples (test set), but whose class is taken into account only for verification of the class assignments. In this work, we use the J48 Java based implementation of C4.5 available in the Weka package [51]. In addition, we performed a large battery of tests with other families of classifiers (function-based, rule-based, naive-based, and Bayesian-based), whose performances are not satisfying and hence not reported. The classification performance is evaluated by computing standard statistical metrics, as accuracy, precision, sensitivity, specificity, and F-measure and by adopting a leave-one-out cross validation sampling procedure [48]. It is worth to note that the classification models can be adopted to classify new subjects whose diagnosis has not been already assessed and that could constitute an independent validation set for further verifying the extracted models.

Finally, in order to prove the validity of the extracted models we performed random permutations of class membership for each classification problem and each signal processing technique (Fourier and Wavelet). We test if our procedure is able to extract meaningful classification models regardless of the class partition imposed on the training set. This would be verified only in the presence of a marked overfitting behavior.

## Results

In this section, we provide the classification results relying on the features extracted with the Fourier and Wavelet Transforms applied on EEG signals of 180 s. Tables 3 and 4 present the results of the Decision Tree classifier considering the Fourier Transform and the Wavelet Transform, respectively.

**Table 3:** Classification performance [%] by using *M*=16 Fourier Coefficients as features and a leave-one-out sampling with 72, 60, 86, 109 folds for HC *vs* AD, HC *vs* MCI, MCI *vs* AD, HC *vs* CASE, respectively

	HC *vs* AD	HC *vs* MCI	MCI *vs* AD	HC *vs* CASE
Accuracy	72.2	71.7	80.2	74.7
Precision	71.1	78.9	80.2	74.0
Sensitivity	72.2	71.7	80.2	74.7
Specificity	59.0	79.0	78.5	46.3
F-measure	71.4	71.8	80.1	74.7

**Table 4:** Classification performance [%] by using *M*=48 Wavelet Coefficients as features and a leave-one-out sampling with 72, 60, 86, 109 folds for HC *vs* AD, HC *vs* MCI, MCI *vs* AD, HC *vs* CASE, respectively

	HC *vs* AD	HC *vs* MCI	MCI *vs* AD	HC *vs* CASE
Accuracy	83.3	91.7	79.1	73.4
Precision	83.3	91.8	79.3	74.7
Sensitivity	83.3	91.7	79.1	73.4
Specificity	78.0	91.5	79.1	51.5
F-measure	83.3	91.7	79.1	74.0

In particular, Table 3 presents the results of the Decision Tree C4.5 classifier concerning the EEG signals with *M*=16 extracted Fourier Coefficients. We obtain 72%, 72%, 80%, 75% of accuracy when dealing with HC *vs* AD, HC *vs* MCI, MCI *vs* AD, HC *vs* CASE classification problems, respectively.

Table 4 presents the performance of the Decision Tree C4.5 classifier concerning the EEG signals processed with the Wavelet Transform. In all classification tasks, the feature extraction based on the Wavelet Transform achieves high classification performance in all metrics, obtaining 83%, 92%, 79%, and 73% of accuracy when dealing with HC *vs* AD, HC *vs* MCI, MCI *vs* AD, HC *vs* CASE classification problems, respectively. In particular, the Wavelet spectral analysis outperforms the Fourier analysis when dealing with EEG signals classification of HC *vs* AD, HC *vs* MCI, and HC *vs* CASE. Conversely, for MCI *vs* AD both signal processing methods lead to comparable classification performances.

Another validation of the proposed procedure is based on the variation of the adopted sampling schema and by applying also a feature selection step with the *Information Gain (InfoGain)* filter as evaluation measure followed by the *Ranker* search method, which reached good performance in our previous study [52]. We perform both 10-fold cross validation sampling and holdout (90% training and 10% test percentage split) combined with Information Gain filter [53] obtaining again satisfying classification performance. We observe an improvement of the HC *vs* CASE and HC *vs* MCI classification tasks both for Fourier and Wavelet transforms reaching even more than 80 and 90% of accuracy, respectively. Conversely, a performance decreasing is observed when distinguishing MCI *vs* AD, probably due to the similarity of the two classes. Classification results are detailed in Table 5.

**Table 5:** Classification performance (Accuracy [%]) by using 10-fold cross validation (CV) sampling and holdout (90% training and 10% test percentage split) for HC *vs* AD, HC *vs* MCI, MCI *vs* AD, HC *vs* CASE, taking into account Wavelet (WT) and Fourier (FT) Coefficients as features

	*Wavelet*		*Fourier*	
	10-fold CV	Holdout	10-fold CV	Holdout
HC *vs* AD	76.4	71.4	80.6	85.7
HC *vs* MCI	93.3	83.3	83.3	83.3
MCI *vs* AD	66.3	88.9	66.7	77.2
HC *vs* CASE	81.7	81.8	84.4	90.9

For validating our results and the extracted classification models we apply the procedure to data with random permutations of class labels. This validation test is performed on 100 different random permutations for each classification problem and for each EEG signal processing technique (Fourier and Wavelet). In particular, when using the Fourier (Wavelet) transform we achieve 56.5% (52.5%), 45.0% (50.5%), 49.5% (45.9%), and 54.5% (50.4%) of average accuracy considering HC *vs* AD, HC *vs* MCI, MCI *vs* AD, HC *vs* CASE permutated classification problems, respectively. Therefore, we obtain an overall average classification accuracy of 50.6%.

We also test other classification methods, such as function-based, rule-based, naive-based, and Bayesian-based (e.g., RIPPER [54], SVM [55], the MultiLayer Perceptron [56]), whose performances are not satisfying and hence are not reported. For instance, we performed a large number of tests with SVMs by tuning the parameters and by setting the complexity, the epsilon for round-off error, the random seed, the tolerance to many different combination of values, but results were not above 65% of accuracy.

Furthermore, we remark that the adopted C4.5 algorithm provides a classification model, a tree built on Wavelet/Fourier Coefficients, from which the investigator can derive the corresponding set of EEG electrodes. Here, the classification trees extracted in our performed analyses mainly involve the electrodes T, O, F, Fp and the wavebands alpha, theta, and delta.

**Fig. 2:**
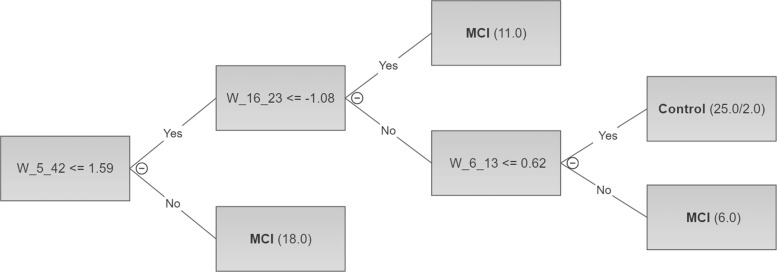
C4.5 tree for HC *vs* MCI of size 7 with 4 leaves. Each path from the root to a leaf represents a classification rule. Each leaf is associated to a class and two numbers. The first number is the total number of instances recognized by the rule, while the second optional number represents how many ones (if any) are misclassified

**Fig. 3:**
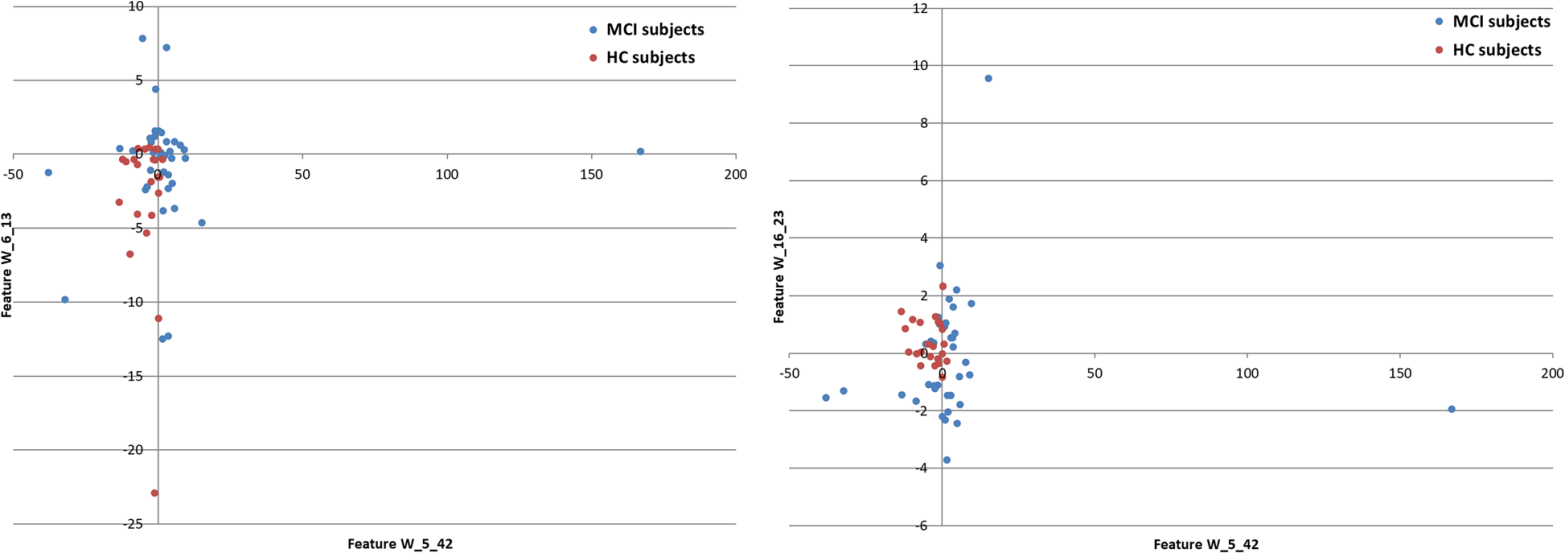
Scatter plot of three example features (i.e., W_5_42, W_6_13, W_16_23) extracted from the C4.5 tree for HC (red points) *vs* MCI (blue points) subjects. The x-axis and y-axis represent the feature values for W_5_42 *vs* W_6_13 on the left, for W_5_42 *vs* W_16_23 on the right

Figure 2 depicts an example of such a tree and Fig. 3 shows the scatter plot of the features extracted from this tree and related to the considered classes (MCI and HC).

## Discussion

Although different neuroimaging techniques (e.g., Magnetic Resonance Imaging, Positron Emission Tomography) can be used for aiding the diagnosis of dementia providing quantitative data about the brain abnormalities [57, 58], EEG is non-invasive, besides being cheaper, simpler and faster to use than other imaging devices [13, 14]. For this reason, automated EEG signal analysis plays an important role in detecting dementia in the early stages, as well as in classifying disease severity [59–61]. Supervised learning is doubtless one of the most popular methods to classify brain disorders with EEG [62–66].

Several studies compared the performance of classification algorithms in terms of sensitivity and specificity, both for the early detection of dementia and for aiding clinical diagnosis. According to [47], our results show that the Wavelet spectral analysis outperforms the Fourier analysis in discriminating EEG of health controls to ones of demented patients. Since EEG may exhibit normal frequency and may appear similar to normal aged control subjects during the earliest stages of dementia [67], the higher accuracy of C4.5 with WT in distinguishing between HC and MCI is a notable result. It is probably due to the fact that WT is suitable for nonstationary signal like EEG that provide linear combination of the sum of wavelet coefficients and mother wavelet with frequency and localization information. In this way, WT is able to detect the slowing of the alpha rhythm, which is more commonly found in intermediate stages of AD [67].

The choice of using three 2-class classification models instead of a single 3-class one is motivated by two main considerations: first, we want to identify if the 3 sets have specific characteristics that single them out with respect to the rest of the data; second, the nature of the adopted classifier is intrinsically binary and therefore they are expected to perform better. Indeed, we obtain poorer performances when 3-classes are used (accuracy below 50%).

Additionally, the overall classification accuracy of 50.6% on 100 different random permutations, for each classification problem and for each EEG signal processing technique, confirms the reliability of our classification models and the absence of over-fitting when considering real classes.

Furthermore, we tested two other sampling schemes (i.e., 10-fold cross validation, and holdout) on the considered classification problems combined with an Information Gain feature selection [53]. The results show a performance decrease when classifying MCI *vs* AD, this can be caused by the similarity of the two stages of dementia. Indeed, Mild Cognitive Impairment become increasingly prone to develop Alzheimer’s or another type of dementia. On the other hand, an improvement of the HC *vs* CASE and HC *vs* MCI classification tasks were observed reaching even more than 80% of accuracy. Notably, distinguishing MCI from healthy control cases can be useful to aid the prediction of the development of later stages of dementia.

In line with most previous studies of EEG classification [23–26], the electrodes mainly discriminant to classify are T, O, F, Fp, whereas the wavebands more recurring are alpha, theta, and delta. After all, an enhanced activity in the theta and delta wavebands, as well as a decreased activity in the alpha and beta ones. Indeed, during cognitive impairment, beta waves (observed in the parietal and frontal region of the scalp) replace alpha waves, whereas theta waves are associated with decreased cognitive activities such as focusing and attention [68].

To conclude, the results on the new and extended data set are improved with respect to our previous study on fewer subjects [34]. Thanks to the Wavelet Transform we obtain promising results also with an enhanced number of HC subjects that are more challenging to discriminate.

## Conclusions

In this work, we proposed an analysis procedure for EEG signals classification of samples affected by neurodegenarative diseases, i.e., Mild Cognitive Impairment (MCI) and Alzheimer Disease (AD), with respect to Healthy Control samples (HC). The analysis is based on a preprocessing phase followed by a feature extraction (Fast Fourier and Wavelet Analysis), and a classification procedure that relies on the well-known supervised learning approach to distinguish the AD, MCI, and HC classes. We tested our procedure on EEG signals recorded on 109 human samples (23 HC, 37 MCI, 49 AD). Through the combination of the Wavelet-based signal analysis and the tree-based classifier C4.5, we effectively identified HC, MCI, and AD experimental samples with better accuracy than the spectral analysis with Fast Fourier Transform.

Finally, we plan to extend the analysis on a new cohort of patients by further increasing the number of considered samples, in particular for the less represented classes, to apply more advanced artifact removal techniques [69], and to study the extracted classification models for identifying further electrodes and wavebands that are related to the investigated diseases.

## Abbreviations

AD: Alzheimer’s disease
DWT: Discrete wavelet transform (DWT)
EEG: Electroencephalography
FFT: Fast fourier transform
FT: Fourier transform
HC: Healthy control
MCI: Mild cognitive impairment
WT: Wavelet transform
